# Dynamics of methanogenesis, ruminal fermentation and fiber digestibility in ruminants following elimination of protozoa: a meta-analysis

**DOI:** 10.1186/s40104-018-0305-6

**Published:** 2018-12-18

**Authors:** Zongjun Li, Qi Deng, Yangfan Liu, Tao Yan, Fei Li, Yangchun Cao, Junhu Yao

**Affiliations:** 1 College of Animal Science and Technology, Northwest A&F University, Yangling, Shaanxi China; 2 State Key Laboratory of Genetic Resources and Evolution, Kunming Institute of Zoology, Chinese Academy of Sciences, Kunming, Yunnan China; 3 College of Pastoral Agricultural Science and Technology, Lanzhou University, Lanzhou, China

**Keywords:** Defaunation, Fiber digestibility, Meta-analysis, Methane production, Rumen fermentation

## Abstract

**Background:**

Ruminal microbes are vital to the conversion of lignocellulose-rich plant materials into nutrients for ruminants. Although protozoa play a key role in linking ruminal microbial networks, the contribution of protozoa to rumen fermentation remains controversial; therefore, this meta-analysis was conducted to quantitatively summarize the temporal dynamics of methanogenesis, ruminal volatile fatty acid (VFA) profiles and dietary fiber digestibility in ruminants following the elimination of protozoa (also termed defaunation). A total of 49 studies from 22 publications were evaluated.

**Results:**

The results revealed that defaunation reduced methane production and shifted ruminal VFA profiles to consist of more propionate and less acetate and butyrate, but with a reduced total VFA concentration and decreased dietary fiber digestibility. However, these effects were diminished linearly, at different rates, with time during the first few weeks after defaunation, and eventually reached relative stability. The acetate to propionate ratio and methane production were increased at 7 and 11 wk after defaunation, respectively.

**Conclusions:**

Elimination of protozoa initially shifted the rumen fermentation toward the production of more propionate and less methane, but eventually toward the production of less propionate and more methane over time.

**Electronic supplementary material:**

The online version of this article (10.1186/s40104-018-0305-6) contains supplementary material, which is available to authorized users.

## Introduction

The rumen provides an ideal habitat for protozoa, whose concentration can reach 10^5^–10^6^ cells/mL. In return, protozoa serve important functions in the rumen microbial ecosystem, such as predation, competition for nutrients, and involvement in symbiotic relationships with other microorganisms [1, 2]. Protozoa prey on bacteria and fungal spores, but are preferentially retained in the rumen, thus reducing the postruminal microbial protein supply [3]. Protozoa compete with amylolytic bacteria for dietary starch, which is mostly fermented into acetate by protozoa [2] while mostly into propionate by amylolytic bacteria [3, 4]. For host animals, the energy recovery efficiency is reduced by 38% when the substrate (glucose) is fermented into acetate but increased by 9% when fermented into propionate [3, 4]. Protozoa are important ruminal hydrogen (H_2_) producers, and the produced H_2_ is mostly converted into methane (CH_4_) by methanogens situated inside protozoa or on their external surface [5–7]. The CH_4_ emissions from ruminants represent 2–12% dietary energy loss [8]. Therefore, the presence of protozoa seems to adversely affect animals’ energy efficiency.

Complete removal of ruminal protozoa, termed defaunation, has been suggested as an efficient method for reducing CH_4_ emissions and enhancing propionate fermentation [9, 10], but these effects have not been consistently observed in studies investigating this 112method [11–13]. Hegarty et al. [12] and Morgavi et al. [13] suggested that the duration of defaunation might be responsible for this inconsistency, but the temporal dynamics of methanogenesis and ruminal volatile fatty acid (VFA) profiles after defaunation are difficult to determine experimentally because of the difficulties in raising defaunated animals over a long-term [14]. Meta-analysis is a statistical method that combines the results from multiple studies to achieve a more precise estimate of treatment effects and to explore the potential sources of between-study heterogeneity [15, 16]. Two prior meta-analyses [14, 17] have summarized the combined responses of rumen fermentation to defaunation; however, the combined effects on ruminal VFA profiles were also inconsistent between them, and neither of them explored the time-dependent effects. Therefore, the current meta-analysis was conducted to quantitatively summarize the temporal dynamics of methanogenesis, ruminal VFA profiles and dietary fiber digestibility in ruminants after defaunation, and to explain the contribution of the defaunated duration to the between-study heterogeneity.

## Methods

### Literature search, screening and data extraction

**Fig. 1:**
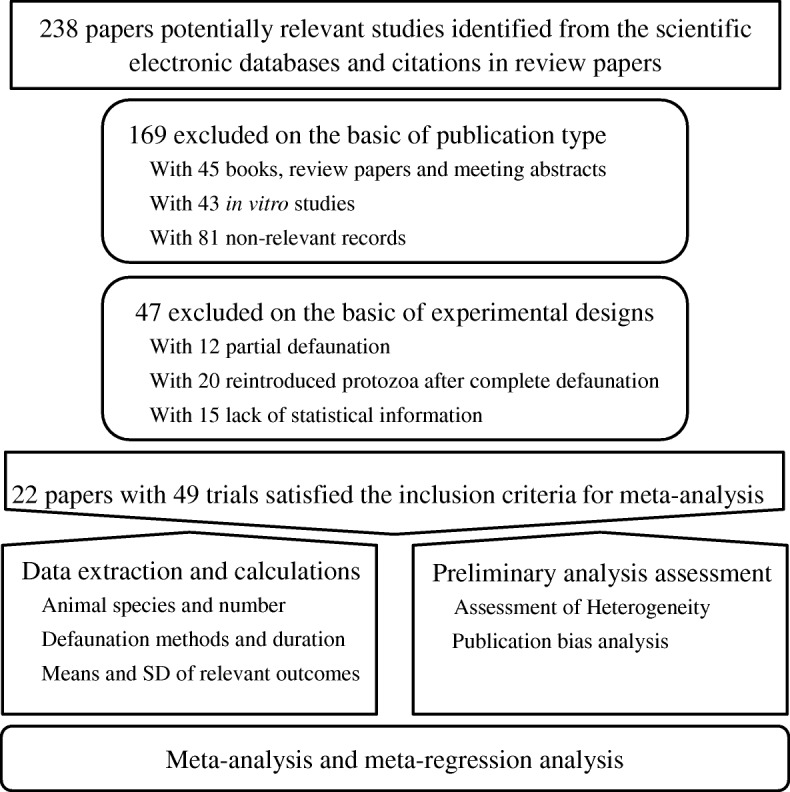
Flow chart of the literature search, screening and data extraction procedures

A flowchart detailing the process of literature search, screening and data extraction is shown in Fig. 1. The inclusion criteria for the studies were as follows: (1) peer-reviewed and published in the English language; (2) complete defaunation in vivo; (3) inclusion of relevant variables for extraction. The relevant variables for this meta-analysis included the daily CH_4_ production, ruminal VFA profiles and dietary fiber total-tract digestibility.

The faunation states of control animals included the natural ruminal ecosystem without any treatment or reintroduction of protozoa after partial defaunation, which appeared to be restored quickly after withdrawal of the protozoa-inhibiting treatment [18, 19]. The control animals into which protozoa were reintroduced after complete defaunation were excluded from the analysis, because preliminary analysis showed that high heterogeneity existed between the faunation and refaunation subgroups (see Additional file 1). Protozoa-free animals were obtained through either the absence of protozoa from birth (BF) or artificial removal of protozoa from the natural ruminal ecosystem (AF). Artificial defaunation was conducted using chemical agents, such as alkanes and sodium lauryl sulfate, or by applying a rumen washing technique. The defaunation duration was calculated based on the schedule of experimental activities. The defaunation duration of BF animals was calculated as their age shortened by 4 wk, because ruminal protozoa did not appear when newborn calves were fed with milk for 30 d [2], and the concentration of protozoa quickly increased after 5 wk of age [20].

The final database included 22 publications, with 49 in vivo studies that satisfied the inclusion criteria for the meta-analysis. Summary descriptions of the selected studies are provided in Table 1. Briefly, among the 49 selected studies, 38 were conducted in sheep, and 11 were conducted in cattle; 18 measured CH_4_ production, 29 measured ruminal VFAs, and 15 measured total-tract fiber digestibility. CH_4_ emissions were measured using the respiration chamber technique or sulfur hexafluoride tracer technique. Methane production was presented in liters per day in most of the studies; thus, values presented in grams or kilojoules (kJ) were converted to liters per day, based on the assumption that one mole of CH_4_ weighs 16 g or contains 890 kJ of energy and occupies a volume of 24.5 L (under conditions of 25 °C and 1 atmospheric pressure).

**Table 1:** Data sources and characteristics of the studies included in the meta-analysis

Source	Trials	Animal	Defaunation duration	Outcomes
Belanche et al. [44]	2	Sheep	23 wk	VFA, digestibility
Bird et al. [11]	2	Sheep	11, 26 wk	CH_4_, VFA, digestibility
Chandramoni et al. [45]	2	Sheep	5, 11 wk	CH_4_, digestibility
Chaudhary and Srivastava [46]	2	Cattle	18 wk	Digestibility
Eadie and Gill [47]	2	Sheep	22, 55 wk	VFA
Eugène et al. [48]	4	Sheep	10, 14, 18, 22 wk	Digestibility
Frumholtz [38]	3	Sheep	5, 26, 52 wk	VFA
Hegarty et al. [12]	4	Sheep	12, 22, 24, 33 wk	CH_4_, VFA
Kasuya et al. [49]	1	Cattle	21 wk	Digestibility
Kreuzer et al. [50]	3	Sheep	9, 10, 11 wk	CH_4_
Morgavi et al. [13]	2	Sheep	6 wk, 2 yr	CH_4_, VFA
Nagaraja et al. [51]	2	Sheep	14 wk	VFA
Nguyen et al. [33]	2	Sheep	9 wk	CH_4_, VFA, digestibility
Ozutsumi et al. [35]	1	Cattle	14 wk	VFA
Santra and Karim [52]	2	Sheep	12 wk	Digestibility
Santra and Karim [53]	3	Sheep	14 wk	Digestibility
Santra et al. [54]	2	Sheep	8 wk	Digestibility
Schönhusen et al. [20]	4	Cattle	4, 5, 6, 7 wk	CH_4_, VFA, digestibility
Sultana et al. [55]	1	Cattle	14 wk	VFA
Williams and Dinusson [56]	2	Cattle	30, 56 wk	VFA
Yáñez-Ruiz et al. [57]	1	Sheep	18 wk	VFA
Zhou et al. [58]	2	Sheep	5 wk	CH_4_, VFA

### Data analysis

The meta-analysis was performed using Stata 14.1 (Stata Corp., Texas, USA).

#### Assessment of heterogeneity and effect size

Between-study variability was quantified via the *I*^*2*^ statistic, which measures the percentage of variation due to heterogeneity [15, 21]. When the *I*^*2*^ value was less than 50%, indicating low heterogeneity, studies were combined using a fixed effects model, which was based on the assumption that the expected effect from each study was homogeneous. When the value of *I*^*2*^ was over 50%, indicating high heterogeneity, studies were combined using a random effects model, based on the assumption that the expected effect from each study was heterogeneous.

The differences in animal species or ages, daily sampling times or dietary forage percentages across the studies caused that the data of certain relevant variables to vary greatly across the studies (Table 2). To reduce these potential interferences, the effect size in this analysis was estimated via the standardized mean difference (SMD), which was calculated as the raw mean difference between the treatment and control groups divided by their pooled standard deviations [15]. For example, although cattle CH_4_ production in the study by Schönhusen et al. [20] was higher than in other subgroup studies involving sheep, it was homogeneous with most of them (Additional file 1: Figures S1 and S2). The studies were weighted using the inverse of the variance of the differences in means. Details of the calculations used in the meta-analysis are provided by Lean et al. [15].

**Table 2:** Data summary and meta-analysis of relevant variables based on all of the selected studies

Variables	No. of trials	Defaunation group			Faunation group			Meta-analysis		
		*n*	Mean	SD	*n*	Mean	SD	*I*^2^, %	SMD	*P*-value
CH_4_, L/d	18	126	20.8	12.4	129	23.4	12.0	71.9	−0.602	0.037
Total VFA, mmol/L	29	211	78.8	29.7	212	87.1	31.3	44.2	−0.549	< 0.001
Individual VFA molar proportion, %										
Acetate	29	210	67.7	5.6	211	66.3	4.3	67.8	0.358	0.083
Propionate	29	210	21.0	4.8	211	20.6	3.1	73.5	0.150	0.515
Butyrate	27	202	8.2	2.0	203	10.2	2.8	68.3	−1.026	< 0.001
A:P	18	78	3.6	1.6	81	3.5	0.7	77.0	−0.284	0.493
Total-tract fiber digestibility, %										
NDF	15	109	55.0	11.6	109	58.1	11.8	55.7	−2.063	< 0.001
ADF	11	82	42.8	2.7	82	45.7	3.7	69.8	−3.075	< 0.001

*n* number of animals, *I*^2^ percentage of heterogeneity across studies, *SMD* standardized mean difference, *A:P* acetate: propionate ratio, *NDF* neutral detergent fiber, *ADF* acid detergent fiber

#### Meta-regression analysis

The meta-regression analysis was performed using the Knapp-Hartung restricted maximum likelihood method [22], with the SMD of the individual studies used as the response variable and the corresponding standard error of the SMD used as the variance. The percentage of between-study heterogeneity explained by the covariate (defaunation duration) was quantified via the adjusted *R*^2^ value.

Preliminary analysis showed that the temporal SMD dynamics after defaunation included a linear phase followed by a plateau phase. The durations of the linear phase and plateau phase for each outcome were dependent on the highest adjusted *R*^2^ of the meta-regression analysis and the minimum *I*^*2*^ of the heterogeneity analysis, respectively; therefore, the two phases might overlap over a short duration. The probability levels were set at *P* <  0.05 for significance and 0.05 ≤ *P* <  0.10 for a trend.

## Results

### Effect size and heterogeneity across all the studies

The meta-analysis based on all the selected studies showed that elimination of rumen protozoa reduced (*P* <  0.05) CH_4_ production, ruminal VFA concentration, the proportion of butyrate and dietary fiber digestibility, and tended to increase (*P* = 0.083) the proportion of acetate (Table 2). However, the heterogeneity across the studies was considerable (*I*^*2*^ > 50%) for most of the responses to defaunation, except for the ruminal VFA concentration (*I*^*2*^ = 44.2%).

### Methanogenesis dynamics during adaptation to defaunation

**Fig. 2:**
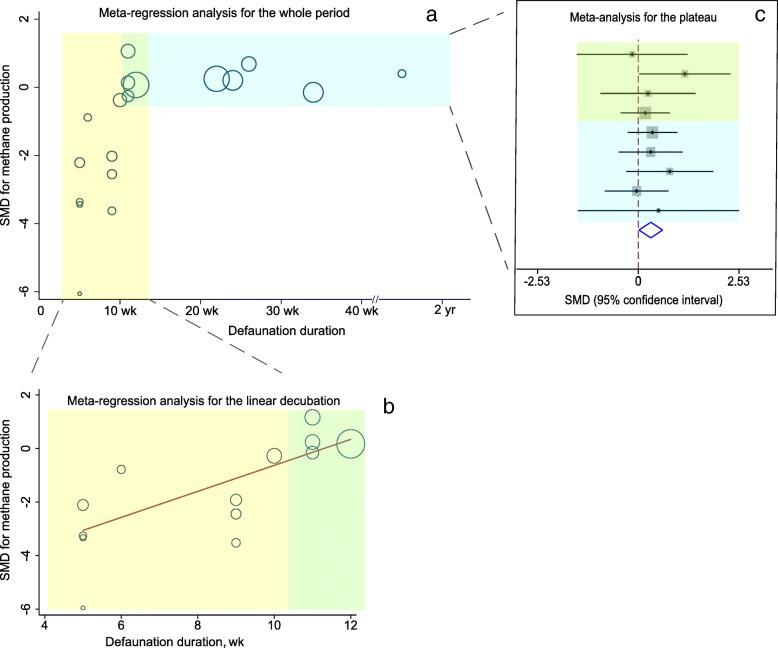
Methanogenesis dynamics during adaptation to defaunation. The temporal SMD (standardized mean difference) dynamics (**a**) after defaunation included a linear phase (≤ 12 wk, yellow portion) followed by a plateau phase (≥ 11 wk, blue portion). The overlap of two phases were the green portion. The linear phase was reanalyzed by meta-regression analysis (**b**), and the plateau phase was reanalyzed by meta-analysis (**c**). Circles in the graph represent the estimates from each study, and the size of the circles represents the percentage of weight of each study. The blue diamond represent in panel (**c**) represents the combined effect and its 95% confidence interval

Compared with that of faunation, the effect size of defaunation on CH_4_ emissions presented a linear relationship over time during the first 12 wk (linear phase) after defaunation (Fig. 2 and Table 3): CH_4_ production was reduced by defaunation (intercept = − 5.484, *P* = 0.003), and the reduction decreased weekly by 0.486 (*P* = 0.003) until 12 wk. The defaunation duration explained 76.8% of the between-study heterogeneity (*I*^*2*^ = 76.2%) during the linear phase, and no between-study heterogeneity (*I*^*2*^ = 0.0%) was observed after 11 wk (plateau phase), suggesting that little fluctuation occurred during the plateau phase. Interestingly, the defaunated animals during the plateau phase presented higher CH_4_ productions (SMD = 0.313, *P* = 0.039) than the control animals.

**Table 3:** Methane emissions, ruminal VFA profiles and total-tract fiber digestibility dynamics during the linear phase and plateau phase

	Meta-regression analysis for the linear phase								Meta-analysis for the plateau phase				
	Duration, wk	No. of trials	*I*^2^, %	Intercept	*P-*value	Coefficient	*P*-value	Adjusted *R*^2^, %	Duration, wk	No. of trials	*I*^2^, %	Pooled effect size	*P*-value
CH_4_	≤ 12	13	76.2	−5.484	0.001	0.486	0.003	76.8	≥ 11	9	0.0	0.313	0.039
TVFA	≤ 12	12	35.5	−1.883	0.008	0.132	0.048	99.9	≥ 11	19	46.7	−0.424	< 0.001
Individual VFA molar proportion													
Acetate	≤ 11	11	76.8	−4.086	0.004	0.563	0.004	76.7	≥ 7	22	47.5	0.748	< 0.001
Propionate	≤ 11	11	84.5	7.306	< 0.001	−0.898	< 0.001	92.2	≥ 7	22	51.2	−0.366	0.033
Butyrate	≤ 11	11	80.6	−7.059	< 0.001	0.732	0.001	92.3	≥ 7	20	54.0	−0.582	0.002
A:P	≤ 11	10	78.6	−6.737	0.001	0.884	0.002	90.8	≥ 7	10	59.2	0.915	0.016
Total-tract fiber digestibility													
NDF	≤ 23	15	55.7	−4.458	< 0.001	0.153	0.005	85.0					
ADF	≤ 23	11	69.8	−6.276	0.001	0.213	0.030	45.1					

*I*^2^, percentage of heterogeneity across studies; *Intercept*, estimate of the effect size after defaunation; *Coefficient*, estimate of the change in effect size per week following defaunation; *Adjusted* *R*^*2*^, percentage of between-study variance explained by the defaunation duration; *TVFA*, total VFA

### Ruminal VFA profiles and total-tract fiber digestibility dynamics during adaptation to defaunation

Consistent with the temporal dynamics of CH_4_ production after defaunation, the ruminal VFA profiles dynamics also included a linear phase (≤ 11 wk) and a plateau phase (≥ 7 wk) (Table 3). After defaunation, decreases in the acetate proportion, butyrate proportion and A:P (intercept = − 4.086, − 7.059 and − 6.737, respectively), and an increase in the propionate proportion (intercept = 7.306) were estimated (*P* <  0.01). These alterations decreased linearly (*P* <  0.01) at different rates over the first 11 wk of defaunation; instead, the ruminal acetate proportion (SMD = 0.748, *P* <  0.001) and A:P (SMD = 0.915, *P* = 0.016) were higher, and the propionate proportion (SMD = − 0.366, *P* = 0.033) was lower in defaunated animals than faunated animals during the plateau phase.

Compared with faunated animals, defaunated animals exhibited a reduced total VFA concentration (intercept = − 1.883 and *P* = 0.008), and the reduction decreased weekly by 0.132 (*P* = 0.048) until 12 wk (Table 3). The duration of defaunation could explain 99.9% of the between-study heterogeneity during the linear phase, although the heterogeneity (35.5%) was low. The decrease in total VFA concentration was still observed (SMD = − 0.424, *P* <  0.001) in defaunated animals during the plateau phase (≥ 11 wk).

Compared with that of faunation, the effect sizes of defaunation on total-tract fiber digestibility were linearly related to defaunation duration (Table 3): total-tract NDF and ADF digestibility (intercept = − 4.458 and − 6.276, respectively) were reduced (*P* <  0.01) after defaunation, and the reductions decreased weekly (*P* <  0.05) by 0.153 and 0.213, respectively, until 23 wk, which was the longest studied duration in the available data.

## Discussion

### Effect size and heterogeneity across all the studies

Based on all the selected studies, this meta-analysis showed that complete elimination of rumen protozoa generated adverse effects on the ruminal VFA concentration, butyrate proportion and dietary fiber digestibility; these findings were consistent with the results of previous meta-analyses [14, 18]. However, the heterogeneity across the studies was considerable for most of the responses to defaunation. Excess between-study variance increases the risk of incorrect average effect sizes when combining studies [15]. For example, the present meta-analysis based on all the studies showed that defaunation tended to increase the proportion of ruminal acetate but had no effect on the proportion of propionate. These findings were consistent with a recent meta-analysis by Newbold et al. [18] but inconsistent with that of Eugène et al. [14], who reported that defaunation induced a reduction in the ruminal acetate proportion and an increase in the propionate proportion. Therefore, the potential source of heterogeneity among the studies needs to be explored to better understand the responses to treatment, and this additional exploration is also one of most important tasks of meta-analysis [15].

### The role of protozoa in rumen carbohydrate metabolism

Despite the fact that protozoa make up a large portion of the rumen biomass, their role in ruminal fermentation and their contribution to the metabolism and nutrition of the host are still topics of substantial controversy [2, 14], due to the difficulty of pure cultivation of protozoa in vitro. Rumen protozoa are not essential to the animal for survival, and defaunation has therefore been used to estimate the role of ciliate protozoa in rumen function. However, the adapted alteration of other microbe after defaunation may interfere with such estimations. Hence, the estimated intercept from the meta-regression analysis more accurately reflected the role that protozoa played in rumen fermentation. Reductions in the ruminal acetate proportion, butyrate proportion and CH_4_ production after complete removal of ruminal protozoa would be expected because protozoa ferment carbohydrates into acetate, butyrate and H_2_ [2], and the H_2_ produced is mostly converted to CH_4_ by methanogens situated inside protozoa or on their external surface [5–7]. A strong correlation between CH_4_ emissions and protozoa concentration has been reported [23], and protozoa-associated methanogens have been estimated to be responsible for 37% of CH_4_ production by ruminants [5]. Additionally, ruminal protozoa possess a full complement of hydrolytic enzymes for fermentation of the major components of feedstuffs, and certain ciliates present a wide range of fibrolytic enzyme genes, ingest small plant particles and use cell wall carbohydrates [18, 24]. Moreover, protozoa can indirectly contribute to ruminal degradation kinetics by maintaining a suitable rumen fermentation environment, for example, by scavenging oxygen to maintain anaerobiosis and slowing the rate of starch fermentation to maintain a proper ruminal pH [24], which favors the development and activity of bacteria and fungi [25, 26]. Therefore, the reductions in dietary fiber digestibility and ruminal total VFA concentration observed in this study would be expected after the complete removal of ruminal protozoa.

### Temporal dynamics during adaptation to defaunation

Although protozoa are important ruminal H_2_ producers and exhibit interspecies H_2_ transfer with methanogens, we found that the effects of short- and long-term defaunation on CH_4_ production were opposite. This finding supports the conclusion of Morgavi et al. [13], who showed that there was not a simple cause-effect relationship between rumen protozoa and methanogenesis. Bird et al. [11] and Hegarty et al. [12] observed higher CH_4_ production (although not significantly so) in long-term defaunated ewes (11 and 26 wk) and lambs (12 to 33 wk) than in faunated animals. The significant increasing effect of long-term defaunation on CH_4_ production detected in this meta-analysis can be attributed to the pooled analysis, in which the number of replicate animals was increased by combining the results of relevant individual studies [15, 16]. The CH_4_ emissions from ruminants contribute to global greenhouse gas emissions and represent energy loss for the animals [8, 27, 28]. Therefore, the potential environmental protection and energy-saving values following defaunation were gradually lost and eventually became negative.

Acetate production during rumen fermentation is accompanied by reducing equivalents ([H]) production, whereas propionate production is accompanied by [H] consumption [29]; the excess [H] is converted to H_2_. The shift in the VFA profiles from propionate to acetate following defaunation increased the H_2_ available for methanogenesis, at least partially explaining the time-related changes in CH_4_ production observed in this study. When glucose is metabolized into acetate, propionate or butyrate, the energy efficiency relative to glucose for animal is 62%, 109% and 78%, respectively [3, 4]. Propionate fermentation is most energy efficient, due to assimilating energy from H_2_ and being the main precursor of gluconeogenesis in animals [3, 30]. In ruminants, the VFAs produced in the rumen satisfy up to 70% of energy requirements [30]. Shabat et al. [31] and Weimer et al. [32] observed that the ruminal total VFA concentration and propionate proportion were higher in highly efficient cows than in cows with low efficiency. Therefore, the decreases in the ruminal total VFA concentration and propionate proportion during the plateau phase also suggested that the elimination of rumen protozoa adversely affected the energy supply of animals in a long run.

The time-related variations in CH_4_, VFA profiles and dietary fiber digestibility implied a series of complex changes in the ruminal ecosystem over the course of defaunation. Nguyen et al. [33] reported that rumen microbes had likely not stabilized after 12 wk of defaunation, which agrees with our results showing that the linear phase for ruminal VFA profiles and CH_4_ emissions lasted 11 wk and 12 wk, respectively. When sudden major changes are made in the diet, it takes approximately 2 wk for the new microbial population balance to become established [34]; the much longer linear phase associated with defaunation suggests that protozoa play an important role in the ruminal ecosystem. Ruminal methanogens appear to develop more slowly than bacteria following defaunation [13]. Hristov et al. [28] noted that reductions in the population of protozoa-associated methanogens might be compensated by an increase in the population of bacteria- or rumen fluid-associated methanogens, and Mosoni et al. [26] found that long-term defaunation (2 yr) increased the abundance of methanogens. In addition, ruminal protozoa elimination results in increased bacterial abundance and changes in bacterial communities [35, 36]; defaunation has been shown to increase the anaerobic fungal population by two fold [37] and the Ruminococcaceae population by six fold [36]. Frumholtz [38] found that long-term defaunation (6 mo) increased the abundance of cellulolytic bacteria. Similar to protozoa, fungi and cellulolytic bacteria are also the main ruminal cellulolytic and H_2_-producing microbes that generate acetate, butyrate and/or H_2_ as primary end products [29, 39]. Therefore, it can be concluded that the increase in the populations of methanogens, fungi and cellulolytic bacteria following defaunation gradually counteracts the defaunation-induced reductions in dietary fiber digestibility, ruminal A:P and CH_4_ production, which may confirm an earlier theory of Weimer [40] indicating that the multiple microbial taxa in the ruminal community show functional redundancy (overlap of physiological function) and may therefore be substitutable with little impact on ecosystem processes [41, 42]. As noted by Taxis et al. [43] regarding the relationship between ruminal ecosystems and function: the players may change but the game remains. These observations also suggest that defaunation is not a good model for estimating the role of protozoa in rumen function due to the compensation effects of fungi and bacteria. Further animal experiments are required to fully understand the succession of rumen bacterial and archaeal community structure and function following defaunation, and the metabolic characteristics of rumen protozoa need be revealed using their genome and transcriptome data.

## Conclusions

The present meta-analysis summarized the temporal dynamics of methanogenesis, ruminal fermentation and dietary fiber digestibility in ruminants after defaunation, and the results showed that defaunation adversely affected dietary fiber digestibility and the ruminal VFAs available to the host animals, although the effects were lessened over time. Furthermore, the energy advantages of defaunation gained by reducing CH_4_ production and shifting ruminal VFA profiles to more propionate were gradually lost over time, and the effects eventually became disadvantageous. Therefore, elimination of rumen protozoa adversely affects the energy supply of animals over the long-term.

## Additional file

Additional file 1: **Figure S1.** Forest plot showing the results of the subgroup meta-analysis of the anti-methanogenic effect size of defaunation, grouped by faunation state and duration of defaunation (11 wk). BF = born and reared protozoa free; AF = artificial defaunation; SMD = standardized mean difference; 95% CI = 95% confidence interval. * I-squared = percentage of heterogeneity across studies; *P*-value of SMD = 0. **Figure S2.** Funnel plot for the effect size of defaunation on CH_4_ production in (A) all studies, (B) short-term defaunation, (C) long-term defaunation, and (D) refaunation. The *P*-value of publication bias is presented. SMD = standardized mean difference, se = standard error. (DOCX 1373 kb)

## Abbreviations

ADF: Acid detergent fiber
AP: Acetate to propionate ratio
NDF: Neutral detergent fiber
SMD: Standardized mean difference
VFA: Volatile fatty acids
