# Supplementary material for: Dynamics of methanogenesis, ruminal fermentation and fiber digestibility in ruminants following elimination of protozoa: a meta-analysis
Source: J Anim Sci Biotechnol. 2018 Dec 18;9:89. doi: 10.1186/s40104-018-0305-6 (PMC6298016; doi:10.1186/s40104-018-0305-6)
Supplement: Supplementary file 1 — Figure S1. Forest plot showing the results of the subgroup meta-analysis of the anti-methanogenic effect size of defaunation, grouped by faunation state and duration of defaunation (11 wk). BF = born and reared protozoa free; AF = artificial defaunation; SMD = standardized mean difference; 95% CI = 95% confidence interval. * I-squared = percentage of heterogeneity across studies; P-value of SMD = 0. Figure S2. Funnel plot for the effect size of defaunation on CH4 production in (A) all studies, (B) short-term defaunation, (C) long-term defaunation, and (D) refaunation. The P-value of publication bias is presented. SMD = standardized mean difference, se = standard error. (DOCX 1373 kb) [file 40104_2018_305_MOESM1_ESM.docx]

**Additional files 1**

**Result and Discussion**

Considering that the duration of defaunation was a factor influencing the effect sizes of defaunation on CH_4_ production, ruminal VFA and dietary fiber digestion among the studies, the studies were grouped into short- and long-term defaunation. The optimal grouping week for the subgroup meta-analysis was dependent on the minimum heterogeneity within the subgroup and the significance level for explaining the heterogeneity across studies. Taking the effects of defaunation on methane emissions as an example, grouping the studies according to the faunation state (faunated or refaunated) and the duration of defaunation (short-term or long-term, by 11 wk) for methane production explained (*P* < 0.001, meta-regression analysis) the heterogeneity across all studies. Neither between-study heterogeneity nor publication bias was observed in the subgroups of long-term defaunation and refaunation (Figures S1 and S2). Therefore, both subgroup meta-analysis (Figure S1) and publication bias analysis (Figure S2) showed that high heterogeneity existed between the faunation and refaunation subgroups. Similar results were observed in terms of ruminal VFA profiles. However, evaluation of the faunation state of the control animals was not the objective of this study; therefore, control animals into which protozoa were reintroduced after complete defaunation (refaunation) were excluded from this meta-analysis. A possible explanation for the heterogeneity between the faunation and refaunation subgroups is that the ruminal ecosystem of refaunation is reshaped by complete defaunation, as discussed in the main text.

***Publication Bias***. Publication bias analysis is performed to test whether selective publication occurs among studies (e.g., if positive results are more likely to be published) (Begg and Mazumdar, 1994). The presence of publication bias was estimated both graphically with funnel plots and statistically using Begg’s test (Begg and Mazumdar, 1994).

**Figure Legends**

**Figure S1.** Forest plot showing the results of the subgroup meta-analysis of the anti-methanogenic effect size of defaunation, grouped by faunation state and duration of defaunation (11 wk).

BF = born and reared protozoa free; AF = artificial defaunation; SMD = standardized mean difference; 95% CI = 95% confidence interval

* I-squared = percentage of heterogeneity across studies; *P*-value of SMD = 0

**Figure S2.** Funnel plot for the effect size of defaunation on CH_4_ production in (A) all studies, (B) short-term defaunation, (C) long-term defaunation, and (D) refaunation. The *P*-value of publication bias is presented. SMD = standardized mean difference, se = standard error.

**References**

Begg CB, Mazumdar M. Operating characteristics of a rank correlation test for publication bias. Biometrics 1994;50:1088–1101.

Belanche A, de la Fuente G, Newbold CJ. Effect of progressive inoculation of fauna-free sheep with holotrich protozoa and total-fauna on rumen fermentation, microbial diversity and methane emissions. FEMS Microbiol. Ecol. 2015;91:fiu026.

Faichney GJ, Graham NM, Walker DM. Rumen characteristics, methane emissions, and digestion in weaned lambs reared in isolation. Aust. J. Agric. Res. 1999;50:1083–1090.

Morgavi DP, Rathahao-Paris E, Popova M, Boccard J, Nielsen KF, Boudra H. Rumen microbial communities influence metabolic phenotypes in lambs. Front. Microbiol. 2015;6:1060.

Nguyen SH, Li L, Hegarty RS. Effects of rumen protozoa of brahman heifers and nitrate on fermentation and *in vitro* methane production. Asian Australas. J. Anim. Sci. 2016c;29:807–813.

Nguyen SH, Hegarty RS. Effects of defaunation and dietary coconut oil distillate on fermentation, digesta kinetics and methane production of Brahman heifers. J. Anim. Physiol. Anim. Nutr. 2016;101, 984–993.

Vermorel M, Jouany JP. Effects of rumen protozoa on energy utilization by wethers of two diets based on ammonia-treated straw supplemented or not with maize. Asian-Austral. J. Anim. Sci. 1989;2:475–476.


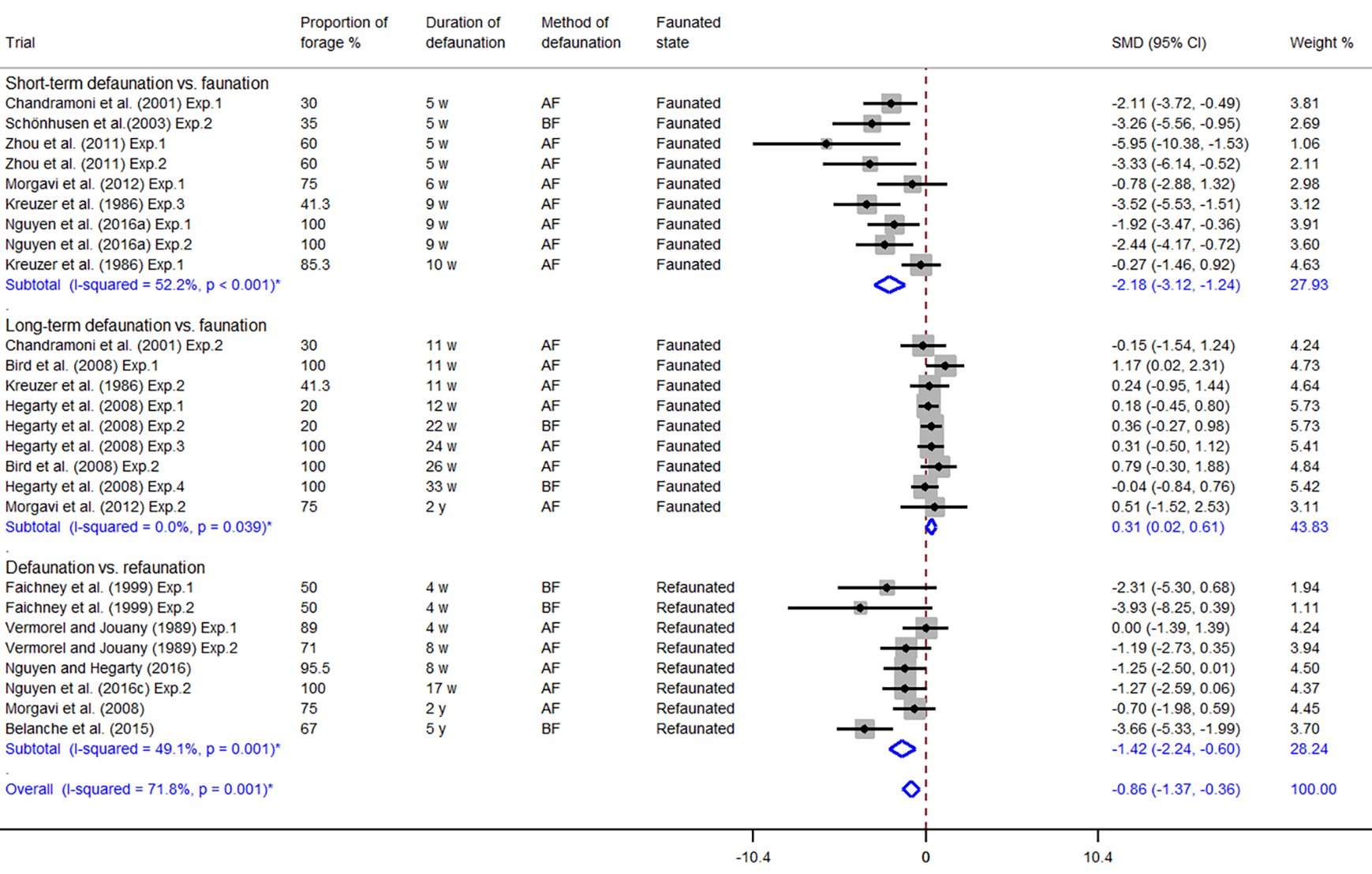
**Figure S1.**


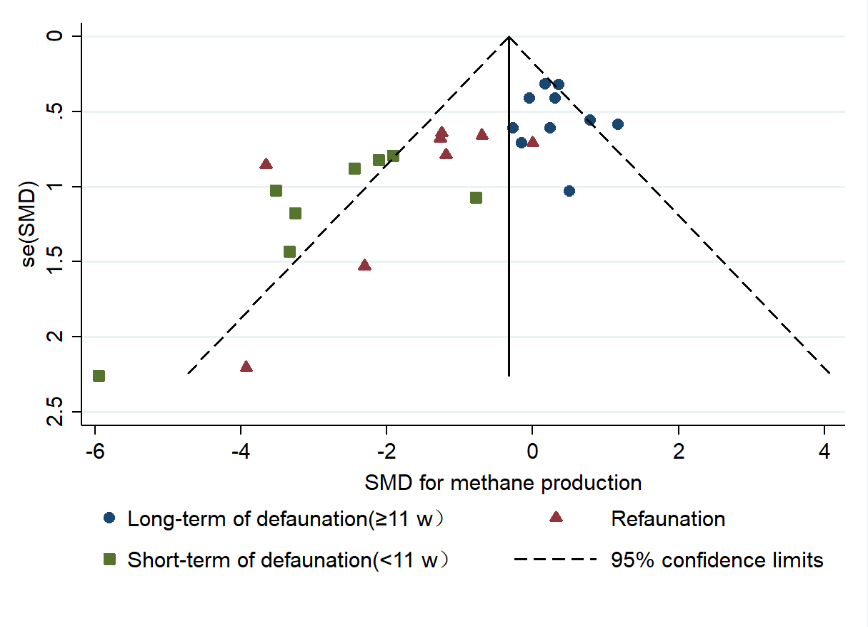

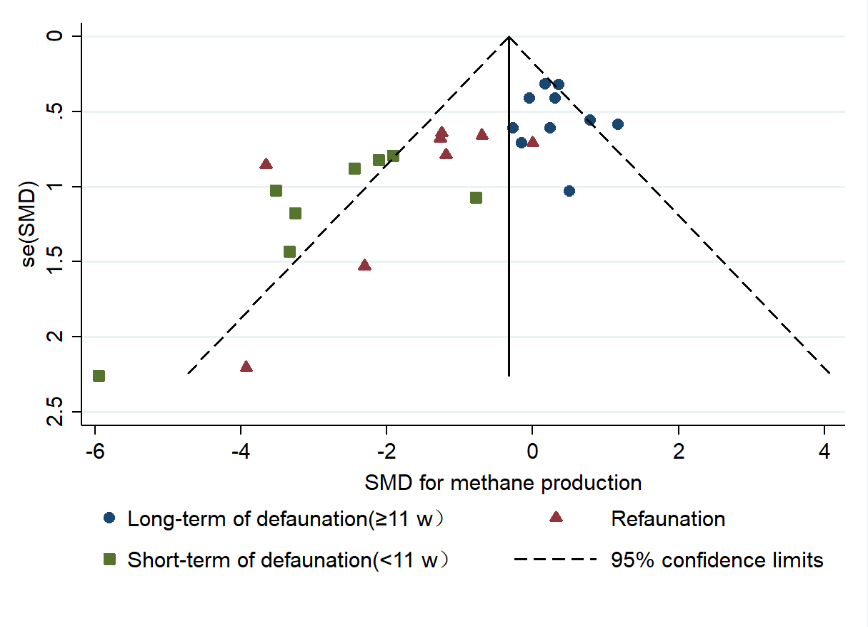


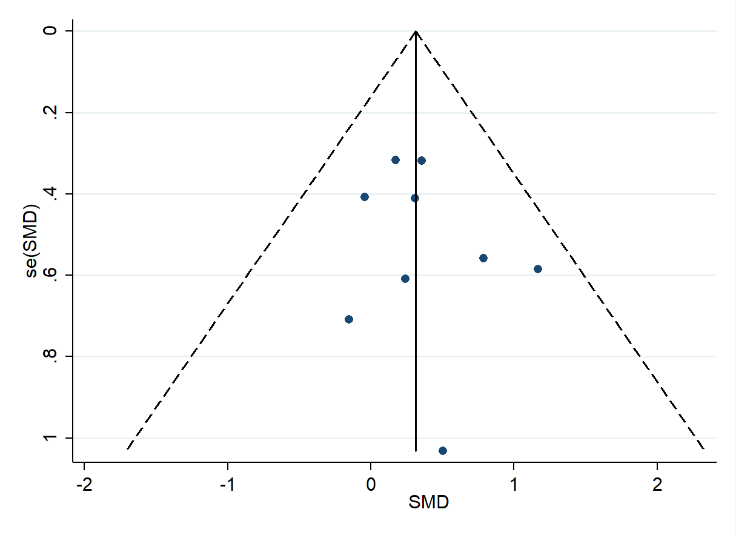


*P* = 0.476

**D**

**C**


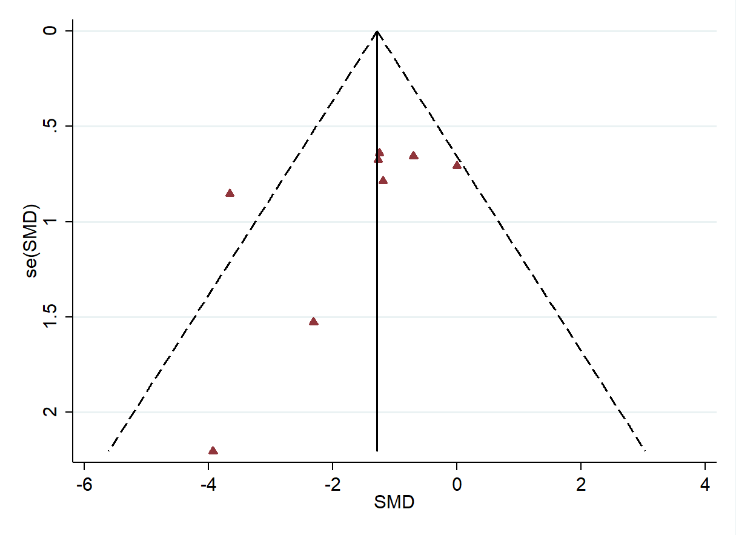


*P* = 0.182

**B**


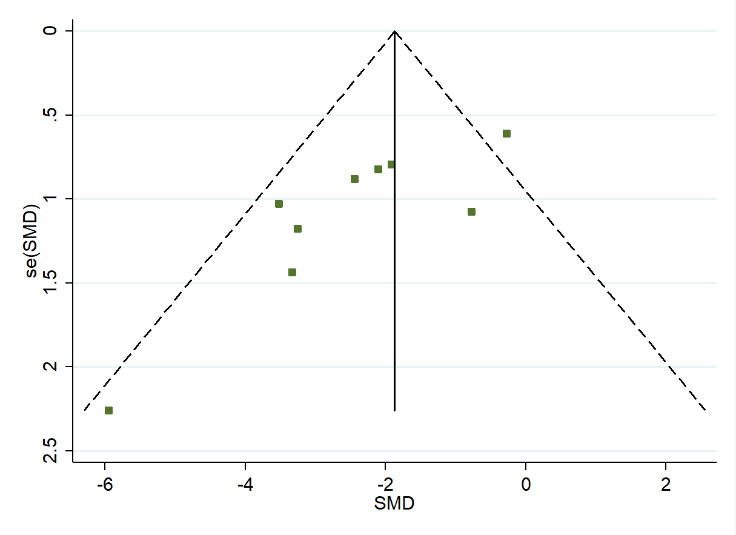


*P* = 0.011


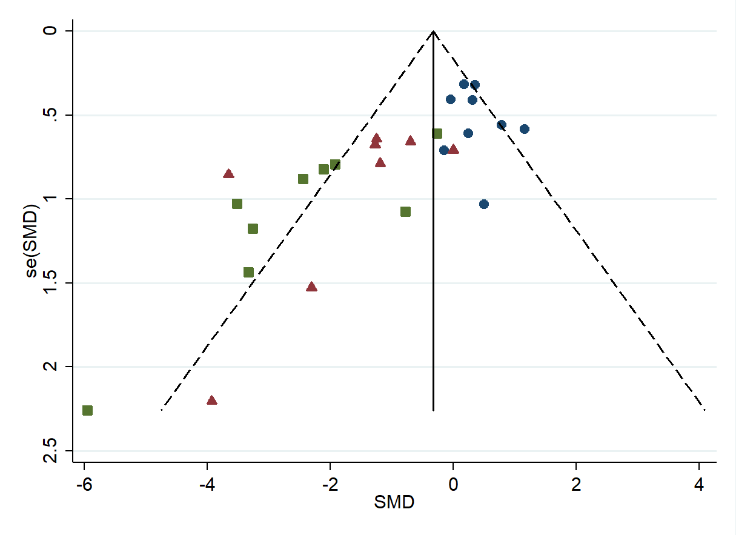


*P* < 0.001

**A**

**Figure S2.**
